# Impaired Cell Cycle Progression and Self-Renewal of Fetal Neural Stem and Progenitor Cells in a Murine Model of Intrauterine Growth Restriction

**DOI:** 10.3389/fcell.2022.821848

**Published:** 2022-07-12

**Authors:** Fu-Sheng Chou, Chu-Yen Chen, An-Chun Lee, Pei-Shan Wang

**Affiliations:** ^1^ Department of Pediatrics, The University of Kansas Medical Center, Kansas City, KS, United States; ^2^ Division of Neonatology, Children’s Mercy-Kansas City, Kansas City, MO, United States

**Keywords:** fetal neural stem cell, cerebral cortex, intrauterine growth restriction, cell cycle, self-renewal

## Abstract

Individuals with intrauterine growth restriction (IUGR) are at an increased risk for neurodevelopmental impairment. Fetal cortical neurogenesis is a time-sensitive process in which fetal neural stem cells (NSCs) follow a distinct pattern of layer-specific neuron generation to populate the cerebral cortex. Here, we used a murine maternal hypoxia-induced IUGR model to study the impact of IUGR on fetal NSC development. In this model, timed-pregnant mice were exposed to hypoxia during the active stage of neurogenesis, followed by fetal brain collection and analysis. In the IUGR fetal brains, we found a significant reduction in cerebral cortical thickness accompanied by decreases in layer-specific neurons. Using EdU labeling, we demonstrated that cell cycle progression of fetal NSCs was delayed, primarily observed in the G2/M phase during inward interkinetic nuclear migration. Following relief from maternal hypoxia exposure, the remaining fetal NSCs re-established their neurogenic ability and resumed production of layer-specific neurons. Surprisingly, the newly generated neurons matched their control counterparts in layer-specific marker expression, suggesting preservation of the fetal NSC temporal identity despite IUGR effects. As expected, the absolute number of neurons generated in the IUGR group remained lower compared to that in the control group due to a reduced fetal NSC pool size as a result of cell cycle defect. Transcriptome analysis identified genes related to energy expenditure and G2/M cell cycle progression being affected by maternal hypoxia-induced IUGR. Taken together, maternal hypoxia-induced IUGR is associated with a defect in cell cycle progression of fetal NSCs, and has a long-term impact on offspring cognitive development.

## Introduction

Survival of extremely low gestational age newborns (ELGAN, those born at less than 28 weeks’ gestation) has increased significantly in the past decade due to the substantial improvement in clinical management and technology (Stoll et al., 2016). However, this increased survival rate also increases the morbidity rate. Surviving ELGANs are at an increased risk for neurodevelopmental abnormalities, including cognitive impairment, learning disabilities, and mental health issues (Botellero et al., 2016, 2017; Costa et al., 2017). Infants who experience intrauterine growth restriction (IUGR) and are born *via* medical induction have the highest risk for adverse neurodevelopmental outcomes (Korzeniewski et al., 2017). The causes of IUGR can be categorized into fetal, maternal, and placental origins, among which placental vasculopathy is considered one of the most common etiologies. Placental vasculopathy is a leading cause of maternal preeclampsia and eclampsia, and can cause placental insufficiency, a condition where blood supply towards the growing fetus is compromised, which results in chronic fetal hypoxia and nutrient deprivation. Imaging studies of human infant brain volumes suggest that decreased volume of the total brain and gray matter is associated with IUGR, but not premature birth (Padilla et al., 2011, 2014). Though imaging studies can shed some light on the mechanism of neurodevelopmental impairment, the detailed cellular and molecular mechanisms underlying impaired cerebral cortex development remain largely unexplored.

It is generally believed that fetal neurogenesis in humans is completed at around 27–28 weeks of gestation. The ELGANs are thus born at an age when fetal neurogenesis is still in progress. Therefore, it is likely that fetal neurogenesis is affected by IUGR in the ELGANs. Key steps in fetal neurogenesis and cerebral cortex development are conserved in mammals (Supplementary Figure S1). In brief, fetal neurogenesis begins following neural tube closure, when neuroepithelial cells first undergo symmetric proliferation to expand its pool size, which is followed by transformation into radial glial cells (RGCs) upon an increase in oxygen tension in the ventricular zone (VZ) coinciding with vascular development (Lui et al., 2011; Lange et al., 2016). RGCs undergo asymmetric cell division to generate intermediate progenitor cells which then undergo multiple rounds of proliferative expansion to populate the subventricular zone (SVZ) before they differentiate into post-mitotic neurons. Newly generated post-mitotic neurons migrate along basal processes of the RGCs to their corresponding layers in the cortical plate (CP) to populate the cerebral cortex (Lui et al., 2011; Chou et al., 2018). The deep-layer neurons are the early-born neurons expressing either Tbr1 or Ctip2 markers. The superficial late-born neurons may express Satb2, Brn2, or Cux1. Temporal transitioning in the generation of layer-specific neurons from RGCs has been proposed to be independent of cell cycle progression of the RGCs. (Gaspard et al., 2008; Okamoto et al., 2016; Ebisuya and Briscoe, 2018)

The association between placental insufficiency-induced IUGR and offspring neurodevelopmental abnormalities does also occur among mammalian species (Hunter et al., 2016). Multiple rodent models of placental insufficiency and IUGR have been developed to assess offspring behaviors and cognitive functioning, with varying results (Hunter et al., 2016). Among published models, uterine artery ligation and hypoxia exposure are the two most frequently adopted approaches to inducing placental insufficiency and IUGR. Using the animal models, the underlying cellular mechanisms to account for neurodevelopmental abnormalities are now beginning to be understood. In a study by Wagenführ et al. (2016), midbrain neurogenesis was found to be hampered in a hypoxia inducible factor-1α (HIF-1α)-independent manner when pregnant dams were exposed to 10% oxygen between E14 and E16. The same group also found reduced mitotic cells in the SVZ, but not in the VZ, of the fetal cerebral cortices following maternal hypoxia exposure between E14 and E16. Correspondingly, cortical neurogenesis and cortical layer thickness are largely influenced by the availability of oxygen during development (Wagenführ et al., 2016). However, it is currently unknown whether cell cycle stages are affected, and what molecular pathways are dysregulated.

In this study, we hypothesized that placental insufficiency-induced IUGR affects fetal neurogenesis in the ELGANs. We aimed to investigate cellular and molecular mechanisms underlying alterations in fetal neurogenesis following exposure to placental insufficiency during critical stages of cerebral cortex development using a hypoxia-induced IUGR mouse model. We first characterized the model that was used in the study, followed by detailing how RGC neurogenesis and cell cycle progression were affected by hypoxia-induced IUGR at the cellular and molecular levels.

## Materials and Methods

### Mice

Timed-pregnant mice were purchased from Charles River Laboratories (Wilmington, WA). All timed-pregnant mice used for this project were mated at a facility where a strict mating time between 6 a.m. and 12 p.m. was implemented in order to achieve similar fetal developmental stages within and across experimental groups. All mice were accommodated at the animal facility of the University of Kansa Medical Center (KUMC) following facility protocols and guidelines. All experiments involving laboratory mice were approved by the Institutional Animal Care and Use Committee (IACUC) at KUMC. Only the CD-1 mouse strain was used for this project. To induce fetal IUGR, timed-pregnant mice at embryonic day (E) 12.5 were placed in a hypoxia chamber (Biospherix, Parish, NY) with 10.5% oxygen concentration for 72 h. All mice were euthanized either with 50 μl Beuthanasia (390 mg/ml) or with carbon dioxide at indicated time points following institutional policies. Experiments were repeated with two or more pregnant dams for quantifications.

### Histology Preparation, Immunofluorescence Staining, and Image Capturing

Embryonic or neonatal cerebral cortices were harvested at the indicated time points and were fixed with 4% paraformaldehyde (PFA) in phosphate-buffered saline (PBS) for 16 h at 4°C, followed by PBS washes three times over a 24-h period at 4°C. Cortical tissues were then cryoprotected with 30% sucrose for a minimum of 16 h on a rocking platform at 4°C, followed by trimming and embedding in 2% low melting point agarose (Fisher Scientific, Hampton, NH). Agarose-embedded tissues were further embedded in Tissue-Tek O.C.T Compound (Fisher Scientific) on dry ice. Cryosectioning was performed on frozen tissue blocks at 20 μm thickness on a Leica CM3050 S Cryostat platform at the KUMC Kansas Intellectual and Developmental Disabilities Research Center Histology Services. Multiple fetal brains were embedded in the same tissue block. Tissue sections were stored at −20°C until use.

**FIGURE 1:**
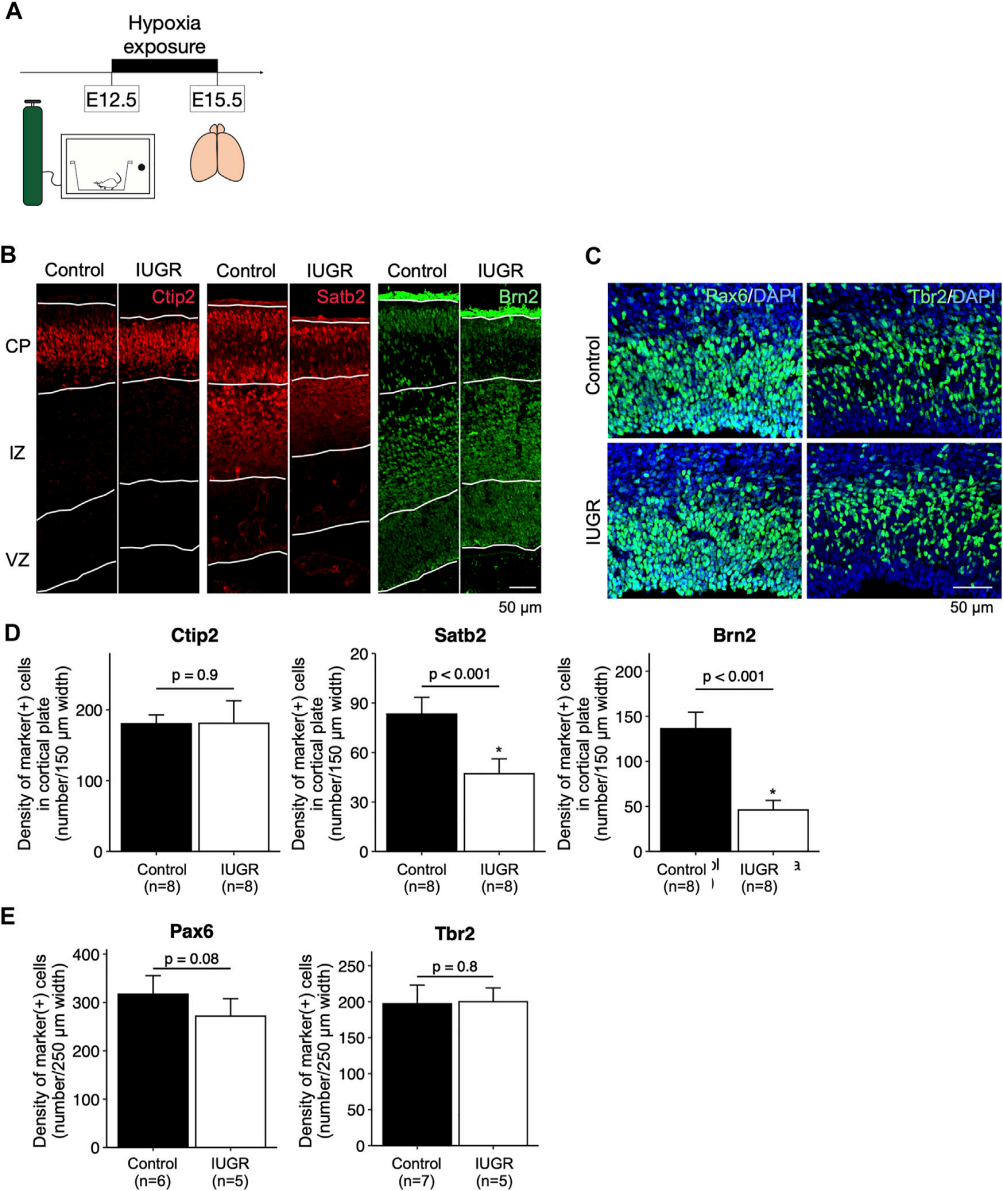
Decreased cortical neurogenesis in the intrauterine growth restriction (IUGR) mouse fetal brain. **(A)** Schematic representation of the experimental design. **(B)** Immunofluorescence staining showing Ctip2-, Satb2-, and Brn2-positive cells in the cortical plate (CP), immediate zone (IZ), and ventricular zone (VZ) in the control and the IUGR groups. **(C)** Immunofluorescence staining showing Pax6- and Tbr2-positive cells in the VZ in the control and IUGR groups. The tissue sections were counterstained with DAPI (not shown) to visualize all cells in a given tissue section. **(D)** Quantification of the densities of cells expressing the indicated markers as shown in Panel B. **(E)** Quantification of the densities of cells expressing the indicated markers as shown in Panel C.

Only brains that remained intact during harvesting were used for immunofluorescent staining, which was performed following protocols as described previously, on brain regions corresponding to the regions as shown in Supplementary Figure S3 (Wang et al., 2012). For EdU labeling of proliferative cells, 1 mg EdU was injected intraperitoneally at the indicated time points. EdU detection was performed by using the Click-iT EdU Imaging Kit (Invitrogen, Carlsbad, CA, United States) following the manufacturer’s instructions. Antibodies used included anti-PH3 (Abcam, 1:100), anti-Pax6 (Biolegend, 1:200), anti-Ki67 (ThermoFisher, 1:200), anti-Tbr1 (Millipore, 1:200), anti-Tbr2 (Abcam, 1:200), anti-Ctip2 (Abcam, 1:100), anti-Brn2 (Santa Cruz, 1:100), anti-Satb2 (Abcam, 1:20), and anti-BrdU (Santa Cruz, 1:100). Nuclei were visualized with 4′,6-diamidino-2-phenylindole (DAPI) counterstaining. Immunofluorescent images were captured using a Zeiss LSM 5 Pascal laser scanning microscope. Image was processed in ImageJ for auto-adjusting of contrast and brightness to facilitate counting. Manual cell counting was performed using the Cell Counter plug-in. Slices from at least three brains were used for quantification. Rectangular areas with the short edge in parallel to the ventricular and cortical surfaces and the long edge spanning across the cortical thickness were cropped for counting (an example may be found in Figure 1B). The regions of the brain where we sampled the staining for cell counting are presented in Supplementary Figure S3. For each experimental comparison, the distance of the short edges was equal among replicates both within each experimental group and between experimental groups. Only one tissue section from each brain was used. As comparable cerebral cortex regions may not be present in the same tissue section across fetal brains in the same tissue block, biological replicates may not always be on the same slice. In other words, replicates may be biological and technical at the same time. Counting was performed by one person (C-YC) blinded to the experimental group.

### RNA Sequencing and Differential Gene Expression Analysis

RNA sequencing (paired-end 75-base pair sequences with a sequencing depth of 25 million reads per sample) was performed by using an Illumina NextSeq sequencer at the Oklahoma State University Genomics facility. RNA integrity was checked by using the Agilent 2200 TapeStation system (Agilent, Santa Clara, CA, United States) prior to sequencing. Post-sequencing data processing, including demultiplexing, file format conversion, and quality trimming were also performed by the sequencing facility. Raw counts were quasi-mapped to mouse cDNA reference sequence using Salmon (0.13.1) (Patro et al., 2017). The Genome Reference Consortium mouse 38 version (GRCm38.p6) was used as the reference genome for mapping. Differential gene expression analysis was performed in R (version 3.6.3) and the RStudio (version 1.2) integrated development environment using the *DESeq2* package (Love et al., 2014). Sequencing raw data has been deposited with the National Library of Medicine, National Center for Biotechnology Information (Accession number: PRJNA817249).

Genes with minimal total raw counts from mapping across all samples of less than 10 were removed before differential gene expression analysis. Raw counts for each gene in each sample were supplied to *DESeq2* for differential gene expression analysis following instructions. Notably, the *DESeq2* package used the median-of-ratios method for the count normalization (Anders and Huber, 2010). We set the threshold of unadjusted *p*-value < 0.05 when considering the statistical significance of differential gene expression given the low sample size and low total reads. For heatmap plotting, we centered the normalized counts across samples to the overall mean and scaled the counts to have the standard deviation be 1.

### Statistical Analysis

Because each embryo has its own placental supply, each individual embryonic brain was considered as one entity in statistical analyses, without taking into consideration the number of the pregnant dams from which the embryos were derived. Each experiment contained 2 to 3 pregnant dams; only one pregnant dam was used in each technical replicate. Each embryonic brain only contributed one tissue section to generate one microscopic view. The *n* indicated in the figures indicates the number of embryonic brains.

Descriptive statistical analysis was performed in R. If not otherwise stated, Student’s *t* test was performed, and a *p* value of < 0.05 was considered statistically significant. If not otherwise stated, bar graphs represent mean, and error bars indicate standard deviation. Gene enrichment analysis was performed using the online Molecular Signatures Database (GSEA, 2022).

## Results

### Antenatal Maternal Hypoxia-Induced Intrauterine Growth Restriction Is Associated With Behavioral Abnormalities

In this study, we aimed to develop a mouse model of antenatal maternal hypoxia-induced IUGR to study its effect on fetal neurogenesis. To this end, we exposed pregnant dams to hypoxia (10.5% O_2_ concentration) between Embryonic day (E) 12.5 to E15.5, a period of time when fetal neural stem and progenitor cells are actively generating various types of early and late neurons (Supplementary Figure S2A). While in the hypoxia chamber, the dams also exhibited reduced food intake (Supplementary Figure S2B), therefore creating combined oxygen- and nutrition-deficient intrauterine environment, fulfilling the clinical characteristics of placental insufficiency. After hypoxia exposure for 3 days, the embryos weighed around 50% (0.39 ± 0.03 g) of the embryos in the control group (0.71 ± 0.08 g) (Supplementary Figure S2C). We allowed pregnancies to continue in ambient air after hypoxia exposure until natural delivery of the pups and found that the weights of the neonatal pups in the hypoxia-exposed group were similar to those in the control group. These IUGR offspring mice in the hypoxia group continued to grow into adulthood. The average weight of the IUGR offspring was significantly higher than that of the control offspring mice at 21 days of life (15.2 ± 1.3 g in control offspring vs. 17.2 ± 1.4 g in IUGR offspring).

To examine neurodevelopmental abnormalities in the IUGR offspring, behavioral tests were conducted. IUGR offspring exhibited a reduction in prepulse inhibition (Supplementary Figure S2D) and were less likely to make complete circles in a Y-maze test, suggesting information processing and working memory deficits, respectively. On the other hand, no abnormalities in social behavior (social arena tests) nor nesting behavior were observed in the IUGR offspring (data not shown). Based on the behavioral test findings, we reasoned that IUGR induced by antenatal maternal hypoxia exposure between E12.5 and E15.5 would be an appropriate model to test our hypothesis. Therefore, we used this model for subsequent experiments to study the underlying cellular and molecular mechanisms.

### Antenatal Maternal Hypoxia-Induced Intrauterine Growth Restriction Resulted in Reduced Cerebral Cortex Thickness

After hypoxia exposure (Figure 1A), the dorsal cerebral cortex of the entire forebrain was significantly thinner compared to the control group, with enlarged lateral ventricles (Supplementary Figure S3). Immunofluorescence staining showed that, in the cortical plate, the density of Satb2-positive and Brn2-positive late-born neurons, but not the Ctip2-positive early-born neurons, were significantly decreased in the IUGR group (Figures 1B,D). In the ventricular zone, the density of Pax6-positive fetal neural stem cells (NSCs) was not significantly decreased in the IUGR group (Figures 1C,E), and the density of the Tbr2-positive progenitor cells were similar between the two groups.

### Neuronal Type Transitioning Was Delayed Following Hypoxia Exposure

**FIGURE 2:**
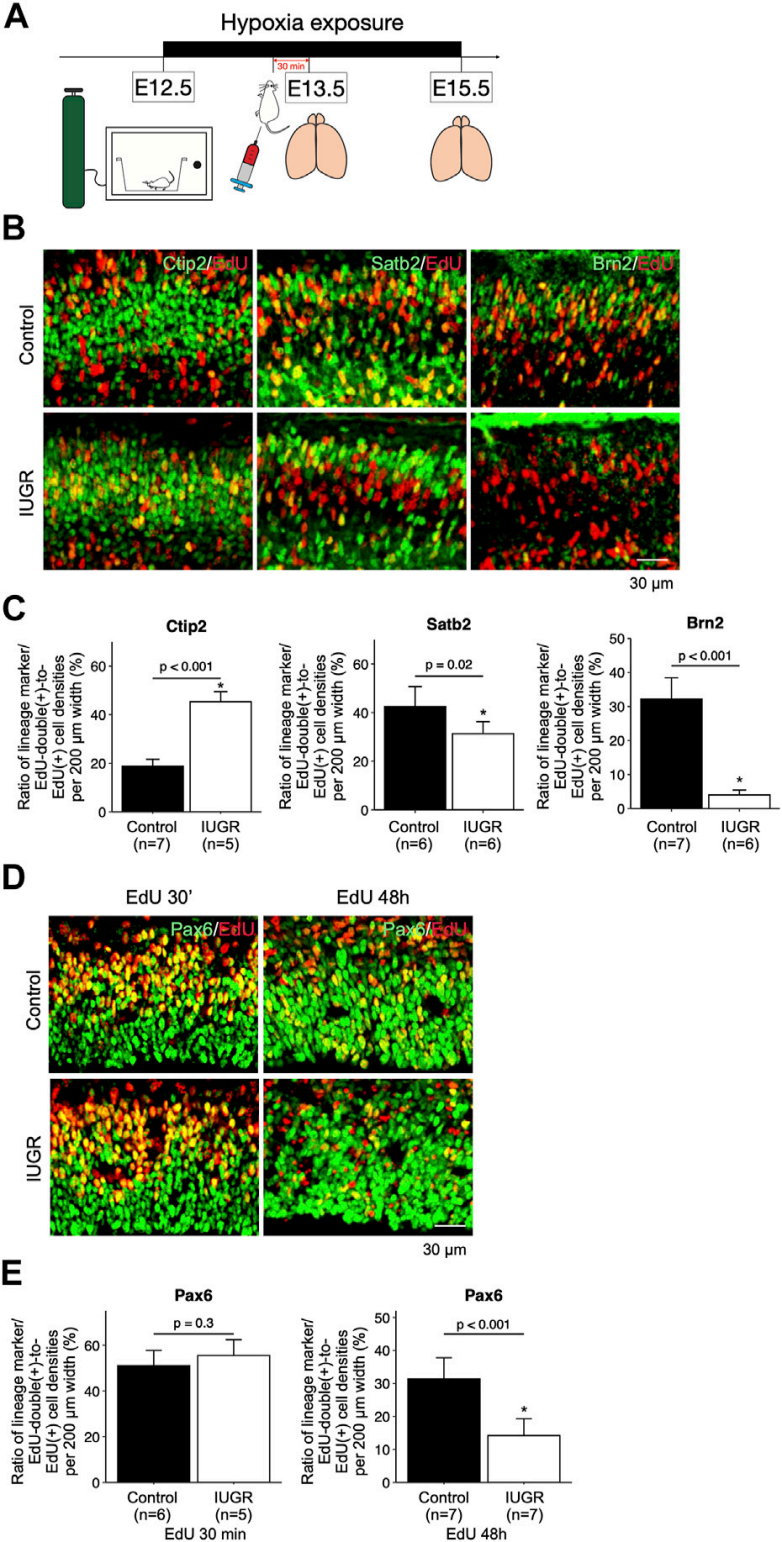
Delayed cortical neurogenesis and decreased number of fetal NSCs in the intrauterine growth restriction (IUGR) mouse fetal brain. **(A)** Schematic representation of the experimental design. **(B)** Immunofluorescence staining showing cells co-expressing EdU and Ctip2, Satb2, or Brn2 markers in the control and the IUGR groups. **(C)** Quantification of the cells in Panel B showing the proportion (shown in percentage) of EdU/lineage marker-co-expressing cell density to total EdU-expressing cell density per view. **(D)** Immunofluorescence staining showing cells co-expressing EdU and Pax6 at the indicated time points in the control and the IUGR groups. **(E)** Quantification of the cells in panel D showing the proportion (shown in percentage) of EdU/Pax6-co-expressing cell density to total EdU-expressing cell density per view.

To test whether cell cycle progression was affected, and to track cell fates of the proliferating fetal neural and progenitor cells, we exposed pregnant dams to hypoxia at E12.5, and injected EdU at E13.5, followed by collecting fetal forebrains at E15.5 (Figure 2A). The forebrains were sliced for immunofluorescent staining with lineage-specific markers including Pax6, Ctip2, Satb2, and Brn2, as well as with EdU. We found significantly more Ctip2/EdU double-positive cells and significantly fewer Satb2/EdU and Brn2/EdU double-positive cells in the IUGR fetal brains (Figures 2B,C), suggesting delayed transitioning of fetal NSCs from producing early-born (Ctip2-positive) to late-born (Satb2-positive and Brn2-positive) neurons. To further investigate the possible cause of this phenotype, we traced the fate of fetal NSCs 30 min and 48 h after EdU injection. The number of Pax6/EdU double-positive fetal NSCs were similar in both control and IUGR groups after 30 min EdU injection. However, the number of Pax6/EdU double-positive fetal NSCs was significantly lower in the IUGR group 48 h after EdU injection, suggesting decreased self-renewal of fetal NSCs (Figures 2D,E).

### Delayed Interkinetic Nuclear Migration of Proliferating Neural Stem Cells is Tied to Delayed G2/M Phase Transitioning in the IUGR Fetal Cerebral Cortex

**FIGURE 3:**
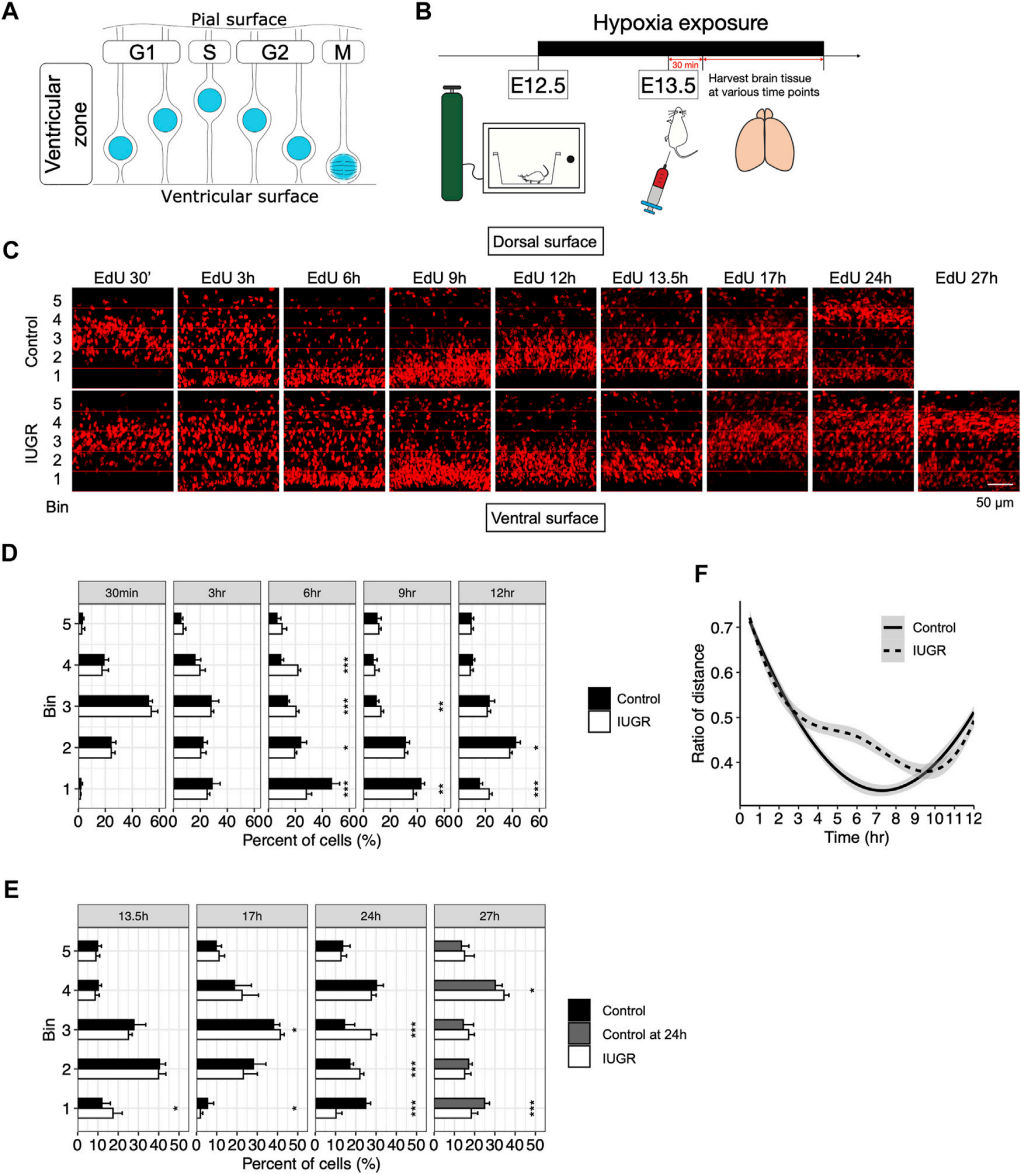
Delayed cell cycle progression of fetal neural stem/progenitor cells in the intrauterine growth restriction (IUGR) mouse fetal brain. **(A)** Schematic representation of the coupling of interkinetic nuclear migration and cell cycle stages. **(B)** Schematic representation of the experimental design. **(C)** EdU staining at indicated time points. Horizontal lines represent boundaries of the bins used for quantification in panels D–F. **(D,E)** Quantifications of the cells in panel C showing the proportion (shown as percentage) of bin-specific EdU (+) cell density to total EdU (+) cell density per view. Note that the bars in the control group for the 27 h plot are the same as those in the 24 h plot. **(F)** Modeling of the distance between the nuclei to the ventricular lining over time in the control and the IUGR group.

Delay in cell cycle progression may be the cause of delayed transitioning of fetal NSCs from producing early-born to late-born neurons. Fetal NSCs were known to progress through the cell cycle with interkinetic nuclear migration within the VZ, with nuclei located away from the ventricular lining during the G1 and S phases; at the beginning of the G2 phase, nuclei migrate towards the ventricular lining, where the M phase takes place. After cell division, nuclei again migrate away from the ventricular lining as cells reenter the G1 phase (Figure 3A) (Miyata, 2008).

To assess interkinetic nuclear migration as a means to track cell cycle progression, we performed a time series study by collecting fetal brains at indicated time points after EdU injection to assess time-lapsed movement of the EdU-labeled nuclei (Figure 3B). As shown in Figures 3C,D, there was no difference in the distribution of the EdU-labeled cells within the VZ at 30 min and 3 h after EdU injection. At 6 h, while most EdU-labeled cells in the control group already migrated towards ventricular lining, EdU-labeled cells in the IUGR group were still dispersed throughout the VZ. At 9 h, the majority of the EdU-labeled cells in the IUGR group were found to have reached the ventricular surface; at the same time, the EdU-labeled cells in the control group that have already reached ventricular surface earlier have started to migrate away from the ventricular surface. At 12 h, most of the EdU-labeled cells in the control group have migrated away from the ventricular surface while some of the EdU-labeled cells in the IUGR group were still at the ventricular surface.

We then measured the distance between EdU-labeled cells and the ventricular lining, presented as ratios between the distance of each EdU-labeled cell to the ventricular lining and the entire thickness of the VZ, and modeled the bulk movement of the labeled cells. We then plotted the ratio of the distance against time and found reduced velocity in nuclear migration towards ventricular surface in the IUGR group with a delay by approximately 3 h (Figure 3F).

At 24 h after EdU injection, we found that EdU-labeled cells in the control group were aggregated separately at the ventricular surface and the dorsal end of the VZ, representing dividing neural stem cells and lineage-committed progenitor cells, respectively. This segregation represents the normal progression of fetal neurogenesis where certain EdU-labeled cells transition from a multipotent stem cell state to a lineage-committed progenitor state, with which cell cycle states are no longer coupled with interkinetic nuclear migration. Such distribution was observed at 27 h after EdU injection in the IUGR group, but with fewer cells at the ventricular surface and more cells at the dorsal end of VZ when compared to the control group at 24 h (Figure 3E), suggesting a skew in cell fate decision following maternal hypoxia-induced IUGR and an accelerated transitioning from a stem to a progenitor state, resulting in a decrease in the stem cell pool size.

### Restoration of Normal Fetal Neurogenesis After Relief From Antenatal Maternal Hypoxia

**FIGURE 4:**
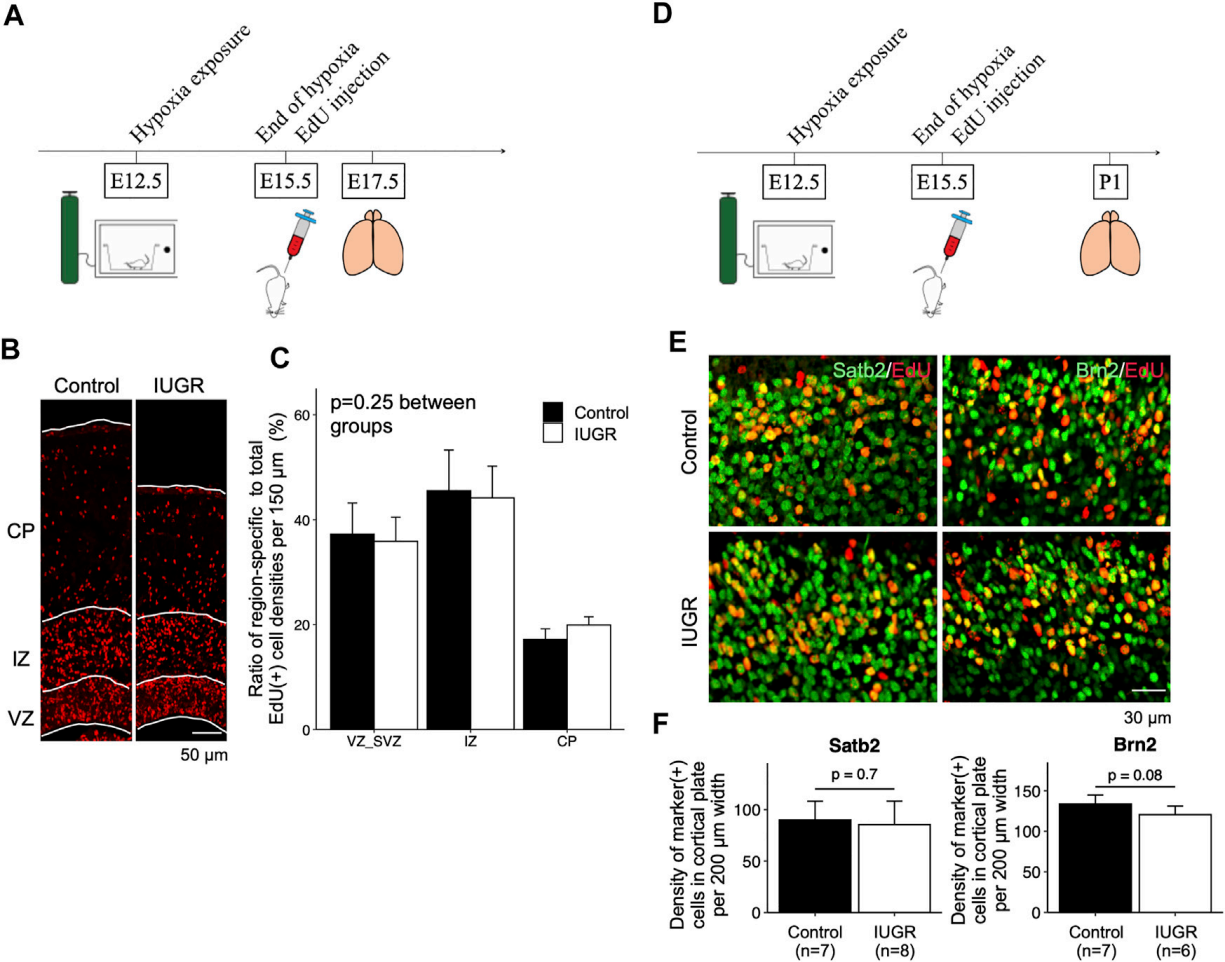
Gestational time-corresponding layer-specific neuron production was resumed following relief from maternal hypoxia exposure. **(A)** Schematic representation of experimental design. Note that brains were harvested on embryonic day (E)17.5. **(B)** Immunofluorescence staining of EdU in the control (*n* = 6) and IUGR (*n* = 6) groups. **(C)** Quantification of the cells in panel B showing the proportion of the region-specific EdU (+) cell density to total EdU (+) cell density per view. A two-way ANOVA test showed no significant difference between experimental groups (*p* = 0.25) but significant difference among brain regions (*p* < 0.001). **(D)** Schematic representation of experimental design. Note that brains were harvested on postnatal day (P) 1. **(E)** Immunofluorescence staining showing cells co-expressing EdU and Satb2/Brn2. **(F)** Quantification of the cells in panel E showing the densities of cells co-expressing EdU and indicated lineage marker.

We then asked whether fetal neurogenesis would resume at a normal pace after relief from antenatal maternal hypoxia. To this end, we labeled proliferating cells with EdU at the end of IUGR induction (E15.5) and placed maternal dams back in ambient air immediately to allow pregnancy to continue (Figure 4A). We then examined the distribution of EdU-labeled cells in the forebrain cortex. We found no differences in the distribution of EdU-positive cells along the path of neuronal migration at E17.5 (Figures 4B,C). We then compared the types of neurons that were generated after IUGR induction to the control group and found that the densities of cells that were double-positive for EdU/Satb2 or EdU/Brn2 were similar between the two groups on postnatal day 1 (Figures 4D–F).

### Multiple Genes Involved in the G2/M Checkpoint and Mitotic Spindle Formation as Well as Self-Renewal Were Dysregulated in the IUGR Fetal Cerebral Cortex

To further investigate early transcriptional response to IUGR induction, we collected fetal forebrain 24 h after hypoxia exposure of the pregnant dams for RNA extraction and sequencing. We found 893 genes that were significantly differentially expressed (unadjusted *p*-value < 0.05) in the IUGR group (Supplementary Figure S4A), 289 of which were genes expressed primarily in the VZ compartment (Fietz et al., 2012). A gene set enrichment analysis using the molecular signatures database found that genes involved in pathways related to hypoxia response (*p*-value < 0.001; false discovery rate < 0.001) and glycolysis (*p*-value < 0.001; false discovery rate < 0.001) were significantly activated (Supplementary Figures S4B,C), suggesting that the cerebral cortices of the IUGR embryos were indeed under the stress of low oxygen tension. Moreover, genes involved in G2/M checkpoint (*p*-value < 0.001; false discovery rate < 0.001) and mitotic spindle formation (*p*-value < 0.001; false discovery rate < 0.001) were also significantly altered. These findings further provide molecular evidence suggesting that G2/M cell cycle phases may be affected by IUGR induction. In addition, genes involved in several pathways that have been shown to play a role in proliferation, differentiation and self-renewal were significantly altered, such as E2F target, mTORc1, Hedgehog and Myc target. Finally, we asked whether gene sets that were differentially expressed in response to low oxygen tension and to nutrient deficiency were different. We exposed pregnant dams to hypoxia (to create low oxygen tension and nutrient deficiency state) or to limited chew supply (to create normal oxygen tension but nutrient deficiency state) from E12.5 to E13.5, followed by fetal brain extraction for RNA sequencing. We showed that a limited number of genes (16 hypoxia-responsive genes and 24 nutrient-responsive genes) were distinctively regulated (Supplementary Figure S5).

## Discussion

In this study, we developed a mouse model of IUGR to study its impact on fetal neurogenesis during cerebral cortex development. We observed delayed interkinetic nuclear migration in proliferating fetal NSCs, which may indicate a delay in G2/M phase progression, although we could not rule out the possibility of cell cycle defects in other phases. Moreover, we found that maternal hypoxia treatment resulted in a decreased fetal NSC pool size and a thinner cortical plate, as well as behavioral abnormalities at a young adult age.

The findings of our study are novel, as we have not come across any human or animal study which characterized the effect of IUGR on cerebral cortex development at the cellular and molecular levels. However, numerous rodent studies using uterine artery ligation or hypoxia exposure of the pregnant dams have established the association between IUGR and impaired cognitive function, recapitulating clinical observations of increased risks of neurodevelopmental impairment among individuals with IUGR and small for gestational age (Dubrovskaya and Zhuravin, 2010; Delcour et al., 2012; Sab et al., 2013; Howell and Pillai, 2014). Similar to our study, a recent study used thromboxane A_2_ to induce IUGR in mice and found deficits in hippocampal dentate gyrus neurogenesis as well as in short-term adult learning and memory (Brown et al., 2021).

Besides cell cycle and self-renewal, temporal identity is the third crucial characteristic of fetal NSCs as the gestation progresses. The temporal evolution of the identities of these primitive cells is likely carried out through a series of epigenetic changes, follows a genetically predefined continual course of action from the start to the end, and is likely one-way. The temporal identity of fetal NSCs has been recapitulated in an *in vitro* model and has been found to be uncoupled from the cell cycle in recent delicate work using a genetic approach (Gaspard et al., 2008; Okamoto et al., 2016). Our observation of delayed transitioning from early-born neuron to late-born neuron production could be either due to a delay in the temporal evolution of fetal NSC identity as a result of IUGR, or a delay in the production of early-born neurons from the neuronal type-committed progenitor cells without a delay in temporal identity evolution. We reasoned that, in the former case, the restoration of the pace of temporal identity evolution should allow progeny cell generation to eventually be as complete as in the control group, with a sufficient final cell number to carry out higher-level cerebral functions. On the other hand, if the latter case stands, the fetal NSCs under IUGR stress may continue the internal evolution of their temporal identity despite not being able to physically produce progeny cells. In the latter model, there would be a permanent loss of neurons. The findings from the post-stress cell tracking experiment appeared to favor the latter model due to a similar pace in the production of post-mitotic neurons and similar neuronal types being produced when compared to the control group at the same gestational period. Future experiments may assess the numbers of various neuronal types in postnatal cerebral cortices and their relative localization and distribution along the cortical span. It is also possible that both models coexist, which will make interpretation much more difficult.

It is encouraging that our findings suggested a G2/M phase delay as a possible cellular mechanism for a reduced cortical mass in the IUGR offspring. Notably, while our cell-labeling data did not differentiate between a defect in the intrinsic G2/M phase progression or cytoskeletal assembly abnormalities as the cause of delayed ventricular nuclear migration, the RNA sequencing results did provide some clues. Specifically, multiple differentially expressed genes encode nuclear proteins required for chromatin assembly and/or DNA breakpoint repair, suggesting that intrinsic dysregulation related to intranuclear events may be responsible for cell cycle defect in fetal neural stem and progenitor cells in response to IUGR induction. Future studies may explore the involvement of each differentially expressed gene (and its associated molecular pathway) in the cause-effect relationship between placental insufficiency and cell cycle defect by rescue experiments to further establish key molecules that may underlie these observations.

Notably, apoptotic cell death could be an additional mechanism for the observed differences in the progenitor pool size between the control and the IUGR fetal brains. Our initial observation did not identify any apoptotic signals in the cortical tissue sections. Future experiments may be warranted to further characterize the roles of programmed cell death in the developing brains.

In conclusion, our data provide cellular insights into alterations in fetal NSC behavior in response to IUGR induction. Cell cycle defects may account for reduced cerebral mass in ELGANs with IUGR and a cellular and molecular basis for their increased risk of adverse neurodevelopmental outcomes.

## Data Availability

The datasets presented in this study can be found in online repositories. The names of the repository/repositories and accession number(s) can be found below: NCBI BioProject—PRJNA817249.
