# Supplementary material for: Impaired Cell Cycle Progression and Self-Renewal of Fetal Neural Stem and Progenitor Cells in a Murine Model of Intrauterine Growth Restriction
Source: Front Cell Dev Biol. 2022 Jul 12;10:821848. doi: 10.3389/fcell.2022.821848 (PMC9314876; doi:10.3389/fcell.2022.821848)
Supplement: Supplementary file 2 [file DataSheet3.PDF]

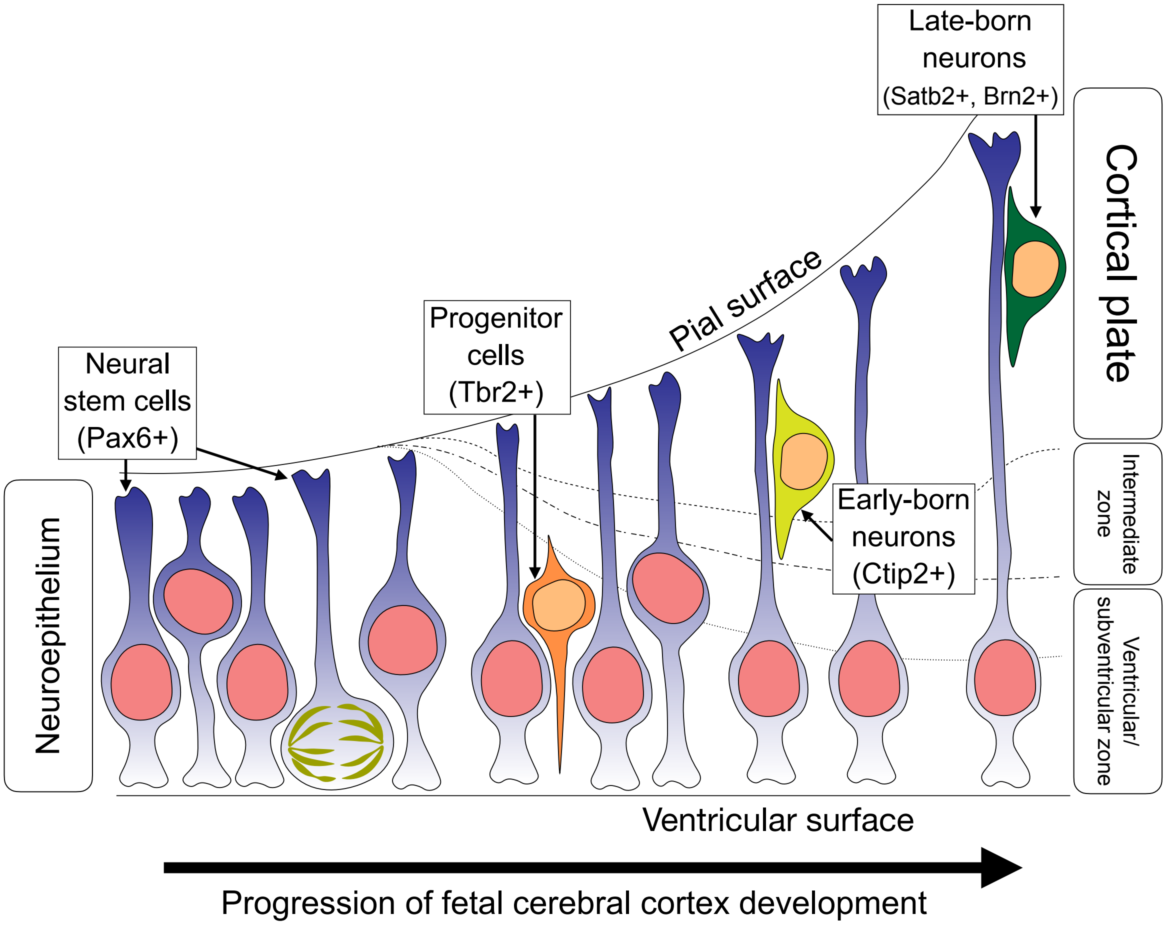

Supplemental Figure 1. Illustration of fetal neurogenesis.

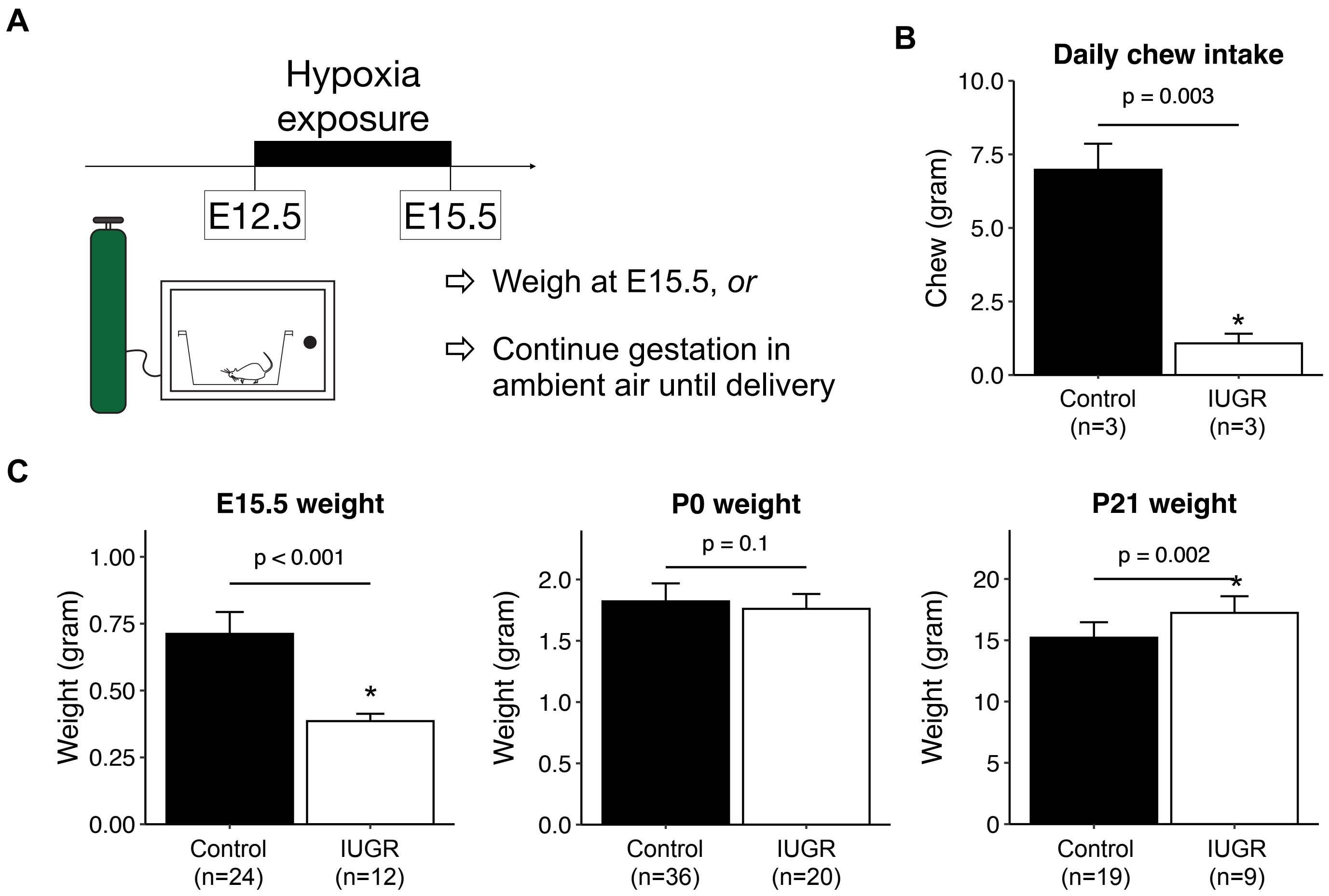

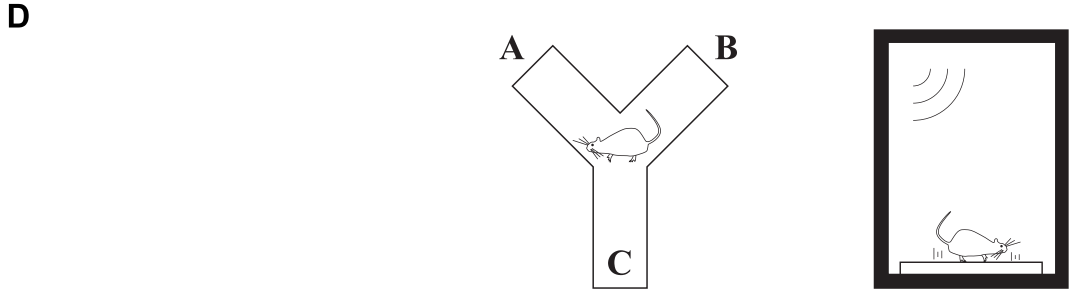

In utero hypoxia exposure	Y maze test - cognitive / memory	Prepulse inhibition - information processing
**Control**	60.1% completed (n = 9)	85.6% inhibited (n = 9)
**IUGR**	40.3% completed (n = 9)	60.9% inhibited (n = 8)
**P value**	0.002	0.004

Supplemental Figure 2. Establishment of a murine model of antenatal maternal hypoxia-induced IUGR. (A) Illustration of maternal hypoxia exposure duration and subsequent experimental plans. (B) The amount of chew consumed by maternal dams (n=3) during hypoxia exposure was measured and compared to maternal dams breathing ambient air (n=3). (C) Weight of embryos on Embryonic Day (E) 15.5, newborn pups on Postnatal Day (P) 0, and P21 juvenile mice with and without *in utero* hypoxia exposure were compared. (D) Offspring mice with and without *in utero* hypoxia exposure at 8 weeks of age were subjected to Y maze and prepulse inhibition behavioral tests.

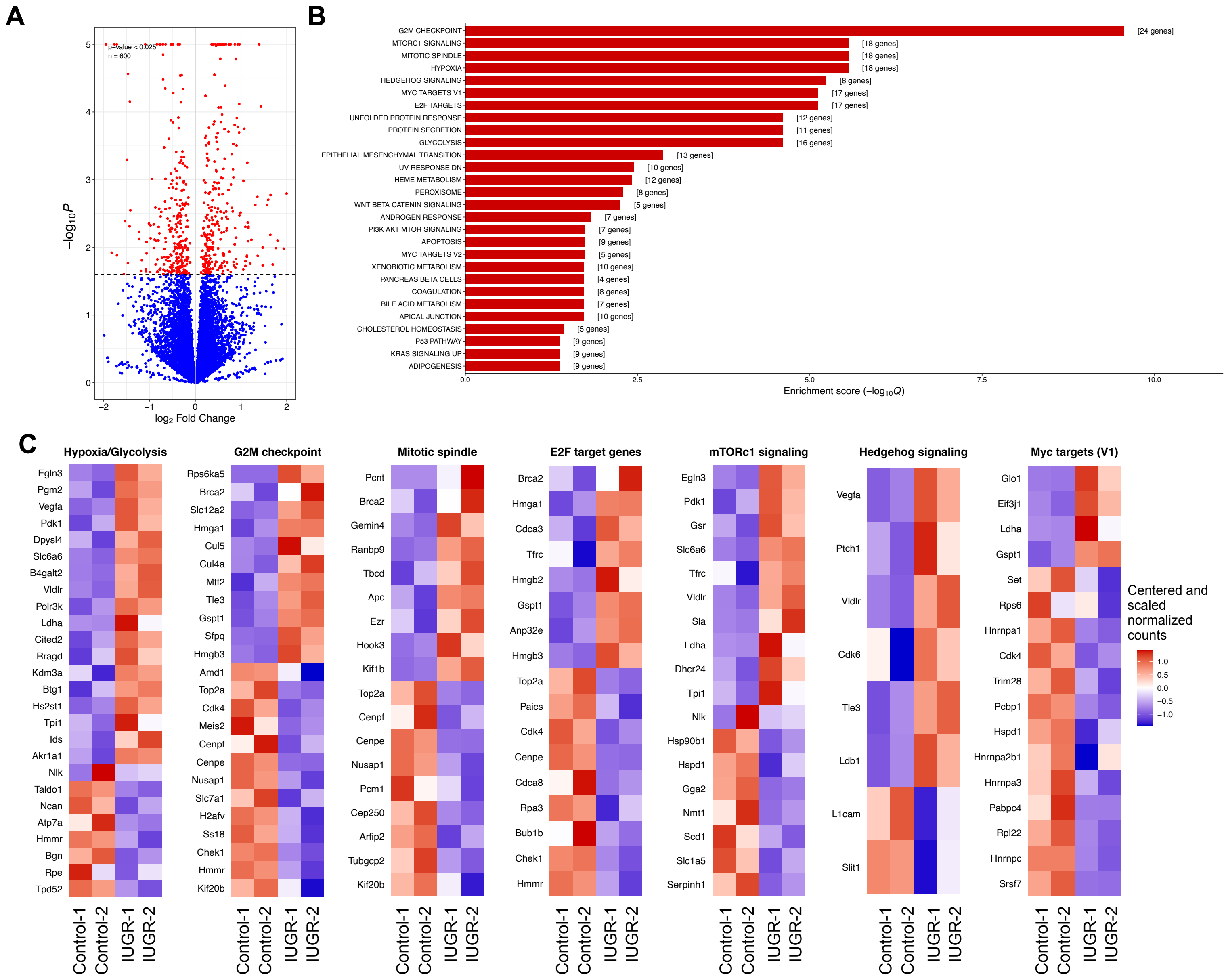

Supplemental Figure 4. Gene expression changes induced by hypoxia-induced IUGR. Pregnant dams were exposed to hypoxia at E12.5 for 24 hrs. Fetal cerebral cortices (n=2 for the control group and n=2 for the IUGR group) were extracted at E13.5 for RNA extraction. (A) Volcano plots showing $\log_2$(fold change) vs. -$\log_{10}$(p-value). (B) A bar graph showing genesets with decreasing statistical significance defined by a false discovery rate adjusted p-value (Q-value) of < 0.05. The Y-axis indicates each geneset name. The X-axis represents -$\log_{10}$(Q-value), a higher number of which indicates a higher likelihood of being a true positive. (C) Heatmaps showing differentially expressed genes in each indicated geneset. Detailed information on RNA sequencing and analysis is available in the text.

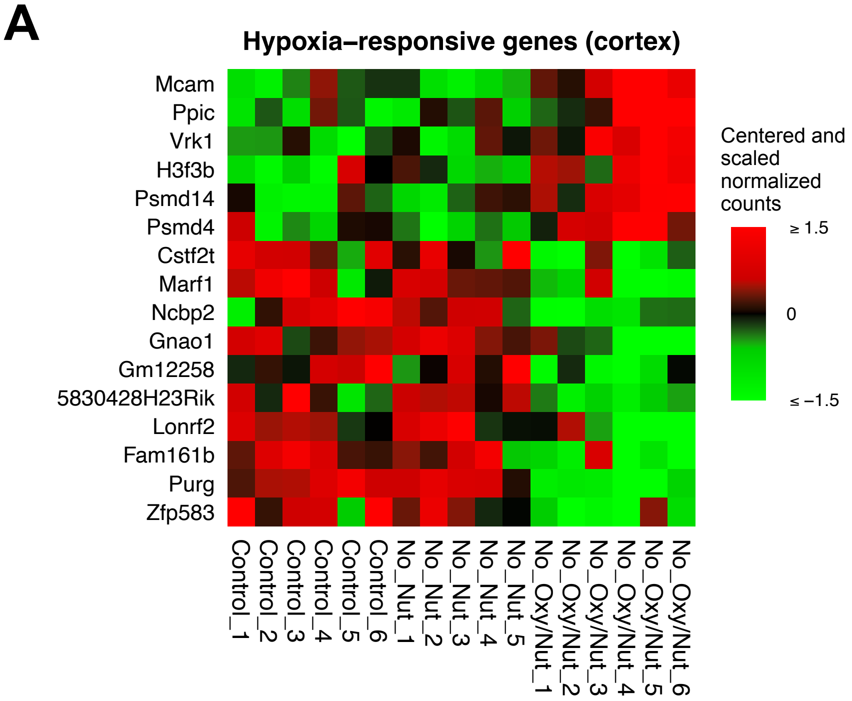

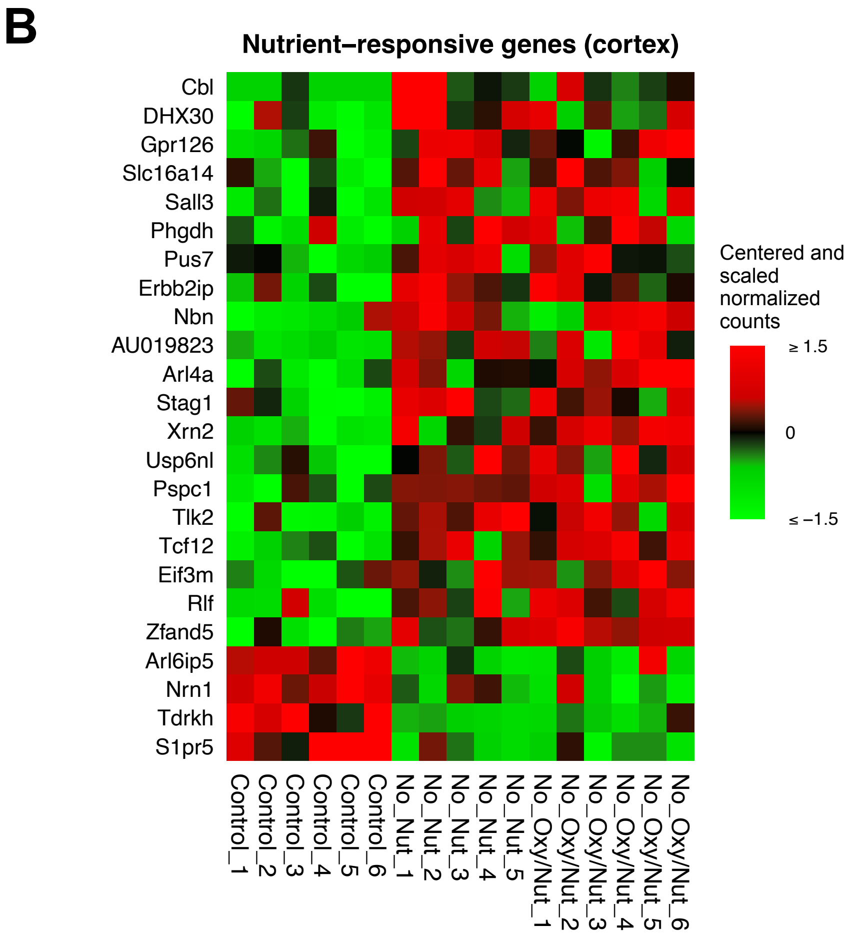

Supplemental Figure 5. Gene expression changes induced by hypoxia or nutrient deprivation. Pregnant dams in the control group (Control_x, n=6) breathed in ambient air with unlimited chow supply. Pregnant dams in the nutrient deprivation group (No_Nut_x, n=5) breathed in ambient air with a daily chow supply limited to 1 gram per day. Pregnant dams in the low oxygen tension and nutrient deprivation group (No_Oxy/Nut_x, n=6) breathed in 10.5% oxygen. The treatment time started at E12.5 and ended at E13.5. Fetal brains were extracted and lysed for RNA extraction and mRNA sequencing. Detailed information on RNA sequencing and analysis is available in the text.
